# SSL: Signal Similarity-Based Localization for Ocean Sensor Networks

**DOI:** 10.3390/s151129702

**Published:** 2015-11-24

**Authors:** Pengpeng Chen, Honglu Ma, Shouwan Gao, Yan Huang

**Affiliations:** 1 School of Computer Science and Technology, China University of Mining and Technology, Xuzhou 221116, China; E-Mails: chenp@cumt.edu.cn (P.C.); cumtmahonglu@163.com (H.M.); 2 Transportation Bureau of Dinghai District, Zhoushan 316000, China; E-Mail: believesea@126.com

**Keywords:** wireless sensor networks, similarity, localization, sea surface

## Abstract

Nowadays, wireless sensor networks are often deployed on the sea surface for ocean scientific monitoring. One of the important challenges is to localize the nodes’ positions. Existing localization schemes can be roughly divided into two types: range-based and range-free. The range-based localization approaches heavily depend on extra hardware capabilities, while range-free ones often suffer from poor accuracy and low scalability, far from the practical ocean monitoring applications. In response to the above limitations, this paper proposes a novel signal similarity-based localization (SSL) technology, which localizes the nodes’ positions by fully utilizing the similarity of received signal strength and the open-air characteristics of the sea surface. In the localization process, we first estimate the relative distance between neighboring nodes through comparing the similarity of received signal strength and then calculate the relative distance for non-neighboring nodes with the shortest path algorithm. After that, the nodes’ relative relation map of the whole network can be obtained. Given at least three anchors, the physical locations of nodes can be finally determined based on the multi-dimensional scaling (MDS) technology. The design is evaluated by two types of ocean experiments: a zonal network and a non-regular network using 28 nodes. Results show that the proposed design improves the localization accuracy compared to typical connectivity-based approaches and also confirm its effectiveness for large-scale ocean sensor networks.

## 1. Introduction

With the development of the economy and technology, the ocean environment is particularly important for human activities related to industry, tourism and urban development. The traditional ocean instruments often suffer from high-cost and time-consuming problems. Wireless sensor networks (WSN) currently play an important role in ocean monitoring [1,2,3], because they can excellently finish the task of real-time monitoring for a long period in large geographical areas. One of the monitoring applications is to deploy sensors on the sea surface for reporting the data. In such networks, many routing and data collection protocols are constructed based on the nodes’ geographic locations. Therefore, the localization of the nodes is the cornerstone of such applications. Furthermore, it is a very challenging problem due to extremely limited resources available at low-cost sensor nodes.

Many excellent ideas have been proposed for sensor localization so far. Generally, there are two types of localization methods: range-based and range-free. The former localization scheme can obtain good localization accuracy. However, it requires either ranging hardware [4,5,6,7,8,9] or system calibration and environment profiling [10]. Hence, it is not appropriate for large-scale ocean monitoring sensor networks. The latter range-free ones localize nodes based on simple sensing, such as wireless connectivity [11,12,13], anchor proximity [14,15,16] or localization events detection [17,18]. These approaches have less cost, but have low accuracy. In fact, a range-free solution with relative distance estimation often has better accuracy and is preferred to be applied into the open scenario. The work in [19] proposes a novel autonomous passive localization approach for road sensor networks, in which the distance between any pair of sensors on roadways is first estimated to construct a virtual graph, and the virtual graph is then matched with the real topology of the road map to identify where sensors are located. It does provide good localization accuracy with low cost, but this method assumes there is no connectivity between nodes and that the road map is known, which is obviously invalid for ocean sensor networks.

This paper is inspired by one of our ongoing projects, in which 18 nodes are deployed on the sea surface for collecting the received signal strength, light and temperature for several months [20]. After analyzing the experimental results, we find that the closer nodes have more similar received signal strength values. Hence, we decide to further extract the embedded position information. Although the received signal strength indicator (RSSI) is generally not considered a good solution for directly estimating physical distance, we argue that it is a free lunch for localization without additional hardware, and it does provide some useful distance-related information [21,22]. Utilizing the RSSI similarity, this paper first estimates the relative distance between neighboring nodes and then calculates the relative distance for non-neighboring nodes with the shortest path algorithm. In the following, the nodes’ relative relation map can be constructed. Given three or more anchors, the nodes’ physical locations are finally obtained through transforming the relative map. Specifically, our major contributions are as follows:

- This paper proposes a novel method to estimate the relative distance between any node pair based on the comparison of RSSI similarity, which has low computation complexity and good distance correlation (the correlation coefficient has increased by about 15% from the experiments).
- A complete localization solution is built for the sea surface sensor network, which fully considers and utilizes the characteristics of both the ocean environment and RSSI values.
- The performance of the proposed design is evaluated by practical experiments with two types of networks, which confirm its effectiveness further.

The rest of the paper is organized as follows: Section 2 surveys the related work. The network model and the motivation are presented in Section 3. Section 4 describes the main design with the algorithm analysis in detail. Section 5 gives the experiment setup and analyzes the experimental results. Finally, we conclude the paper along with future work in Section 6.

## 2. Related Work

Existing localization methods can be roughly categorized into two classes: (i) range-based [23,24,25,26,27]; and (ii) range-free localization [28,29]. The below will give the detailed description and analysis about the two approaches.

Range-based solutions estimate absolute distances or angles among nodes and then apply triangulation or multi-lateration for location calculation. The representative algorithms are RSSI [22], angle of arrival (AOA) [7], time of arrival (TOA) [8], time differences of arrival (TDOA) [9], and so on. The RSSI scheme employs some empirical models to estimate the distance based on the loss of power, which only relies on the communication module and, hence, has low hardware cost. The AOA approach estimates nodes’ locations by measuring the direction from which a signal is received. The TOA and TDOA methods measure the propagation time and calculate the distance based on the propagation speed. Because the ultrasound signals usually propagate only a very short distance, TDOA is not quite suitable for ocean sensor networks. In all, the above methods achieve good accuracy by virtue of additional and costly hardware, which are not suitable for large-scale ocean monitoring systems. Although there are also some RSSI-based localization methods with noise filtering or fingerprint matching, they have to accomplish environment profiling and calibration, which are also impractical for large-scale ocean sensor networks.

Range-free solutions, like approximate point-in triangulation (APIT) [15] and the centroid method [16], do not need to measure accurate distances or angles. They make use of the proximity information to anchor nodes. For instance, APIT employs an area-based approach to finish the location estimation by dividing the environment into triangular regions. Through combining multiple positions of anchors, the possible area in which a node resides can be reduced. Concurrently, connectivity-based protocols, such as distance vector-hop (DV-Hop) [30], multi-dimensional scaling-map (MDS-MAP) [31], and so on, construct hop-based distances for large-scale sensor network localization using neighborhood sensing. In the DV-Hop scheme, anchors flood their location throughout the whole network in which each node maintains a running hop count. Nodes calculate their positions based on the anchor locations, the hop count and the average distance per hop. In those systems, only a small number of anchors is needed, which significantly reduces the system cost. In addition, a modified extended Kalman filtering technique [32] is proposed for tracking applications with insufficient and intermittent observations. By deeply analyzing existing protocols, we find that connectivity-based localization schemes do not make full use of the information available from neighborhood sensing.

Considering the limitations of previous works, this paper presents a novel similarity-based localization approach especially for wireless sensors deployed on the sea surface, which has a better performance than state-of-the-art connectivity-based methods.

## 3. Motivation

**Figure 1:**
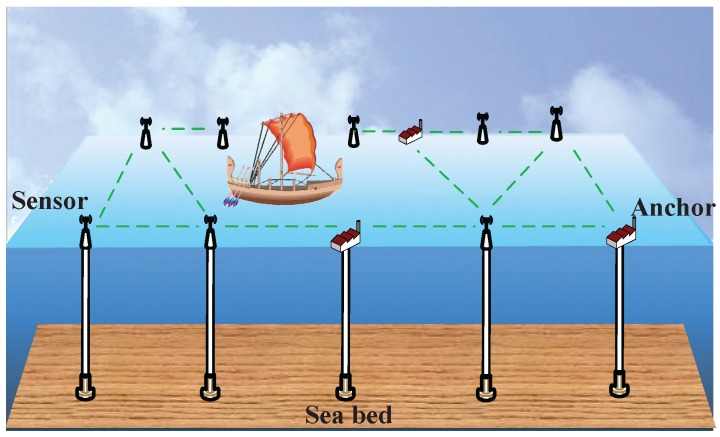
Network scenario.

A network model composed of several anchors and static nodes with unknown locations is considered. All of the sensors are deployed on the sea surface and are used to monitor the ocean environment or the ships’ information on the sea route, as shown in Figure 1. The sensor nodes are fixed to keep stationary or anchored to the sea bottom by ropes. In this context, there are many requirements and constraints for node localization as follows:

- The localization algorithm must be energy efficient, because the replacement of the batteries is often troublesome. This requires that there is less communication and lower complexity.
- The localization algorithm must be robust, since the aggressive and rugged ocean environment may cause the loss of nodes or node faults.
- The localization system should be low cost. The monitored ocean area often needs many nodes to cover. Thus, we cannot add too much additional equipment (e.g., GPS) for localization.

Realizing the above requirements, the RSSI-based method is preferred to be utilized to perform the localization. Unlike time-based solutions, it does not need additional hardware and communication. However, it is hard to accomplish the environment profiling and calibration, due to the unpredictable environmental factors. Besides, one RSSI value may correspond to multiple different distances. Hence, directly using the RSSI values and empirical models is not reasonable for node localization.

Although a network-wide monotonic relationship between RSSI and physical distance does not hold, the single-node monotonic RSSI-distance relationship holds well in an open scenario [33,34]. Our experimental data also confirm that. The RSSI values of two nodes can indeed reflect the “near-far” relationship between them. However, this inherent correlation is not fully utilized by previous typical connectivity-based localization methods. They only consider the binary connection information, leading to a degraded localization accuracy. This forms the motivation for our work, which is to explore the distance and position information embedded in the RSSI values for improving localization accuracy.

## 4. Signal Similarity-Based Localization

### 4.1. Distance Estimation for Neighboring Nodes

This subsection presents the main design of a relative distance among one-hop neighbors through comparing the similarity of the RSSI. Without loss of generality, we describe the calculation process using nodes *i* and *j* step by step.

In order to estimate the near-far relation between nodes, a reference object must be assigned. Here, we construct a reference set $R_{ij}$ for the node pair (i,j), where *j* is one of the neighbors of node *i*. Let $S_{i}$ and $S_{j}$ denote the neighbor sets of nodes *i* and *j*; the reference set can be constructed as $R_{ij}=S_{i}\cup S_{j}\cup \left\{i\right\}\cup \left\{j\right\}$, and the number of elements in $R_{ij}$ is *m*.

Given the reference set $R_{ij}$, we can then generate two vectors $V_{i}$ and $V_{j}$, respectively, for nodes *i* and *j* based on the following vector generation rules. Both $V_{i}$ and $V_{j}$ consist of RSSI values from all of the nodes in $R_{ij}$. Namely, $V_{i}=\{RSSI\left(i,n_{1}\right),RSSI\left(i,n_{2}\right),\cdots ,RSSI\left(i,n_{m}\right)\}$, where $n_{l}\in R_{ij}$ and l=1,2,⋯,m.

Vector Generation Rule 1: The “RSSI” itself is assigned as its sending power. For example, if the sending power of the node *i* is 0 dBm, we have RSSI(i,i)=0 in the vector $V_{i}$.

Vector Generation Rule 2: For the node *k* not in the set $S_{i}$, but in $R_{ij}$, RSSI(i,k) is assigned as -100 dBm (the receiver sensitivity threshold) in the vector $V_{i}$.

Obviously, for a node pair *i* and *j*, the vector $V_{i}$ contains the RSSI values of the node itself, its one-hop neighbors and some non-neighbors, which belong to neighbors of node *j*. We can further observe that the vector has two important features. First, it is position dependent and not unique for a node. The generated vector $V_{i}$ is different for node pairs (i,j) and (i,k) for any j≠k. Second, it is obtained without ranging. Hence, the vector embeds the location information on both connectivity and proximity with a low-cost method, which is suitable for relative distance estimation.

Now, the problem of relative distance estimation between *i* and *j* is turned into the similarity calculation problem of two vectors $V_{A}$ and $V_{B}$. In this paper, we use the Manhattan distance of the two vectors as their relative distance. The Manhattan distance is the distance between two points measured along axes at right angles. It can be easily generalized to higher dimensions, which is illustrated below. Given two vectors $\left(X_{1},X_{2},X_{3},\cdots \right)$ and $\left(Y_{1},Y_{2},Y_{3},\cdots \right)$ with the same number of elements *N*, the Manhattan distance of two vectors is:

(1) $$MD=\sum_{k=1}^{N}\left|X_{k}-Y_{k}\right|$$

From a computational perspective, the Manhattan distance costs few resources and has obvious advantages. Moreover, it can effectively reflect the difference of the received signal. The experimental results also confirm that Manhattan distance has better accuracy than the classic Euclidean distance. Below, the terminology SSLM (SSL with the Manhattan distance) is used to represent the relative distance estimated with the Manhattan distance, while SSLE (SSL with the Euclidean distance) indicates the relative distance calculated by the Euclidean distance.

**Figure 2:**
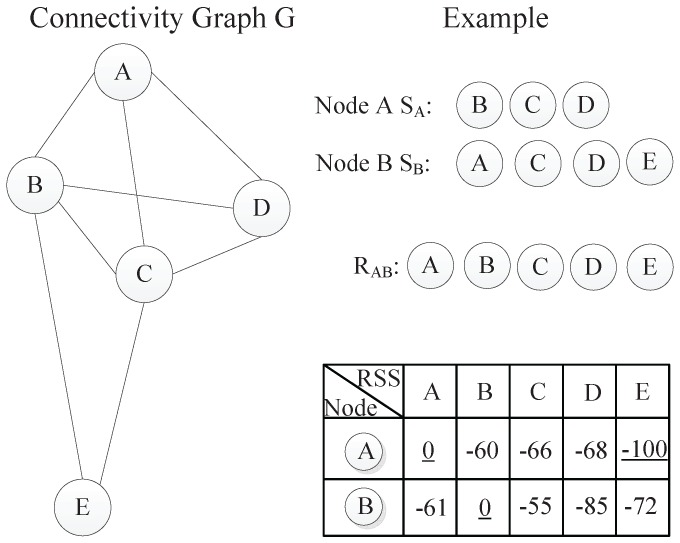
The calculation process of RSSI similarity.

A simple example is shown in Figure 2. On the left of the figure, the connectivity of the network is illustrated through a graph *G*. Additionally, the reference set $R_{AB}$ and RSSI values are listed on the right. Next, we will elaborate the calculation process of the relative distance based on this example. The reference set $R_{AB}$ of $S_{A}$ and $S_{B}$ can be easily obtained, which is {A,B,C,D,E}. According to the proposed rules, the vectors $V_{A}$ and $V_{B}$ are set as [0,-60,-66,-68,-100] and [-61,0,-55,-85,-72], respectively. Then, the SSLM value SSLM(A,B) between *A* and *B* is |0+61|+|-60+0|+|-66+55|+|-68+85|+|-100+72|, which is 177 namely.

With the RSSI values, the relative distance of any two neighboring nodes can be easily obtained. Furthermore, we can see that the SSLM(A,B) is equals to SSLM(B,A). This feature can reduce the calculation times of the relative distance from $n^{2}$ to n(n+1)/2, where *n* is the total number of nodes in the network. Moreover, SSLM can achieve a sub-hop resolution. In the traditional hop-based approach, all neighboring node pairs have an identical hop distance of one, while the SSLM distances between neighboring nodes are different and determined by RSSI values.

### 4.2. Distance Estimation for Non-Neighboring Nodes

In this subsection, the relative distance estimation for non-neighboring nodes is considered. We define the smallest accumulated SSLM along a path between two nodes as the relative distance of non-neighboring nodes.

**Figure 3:**
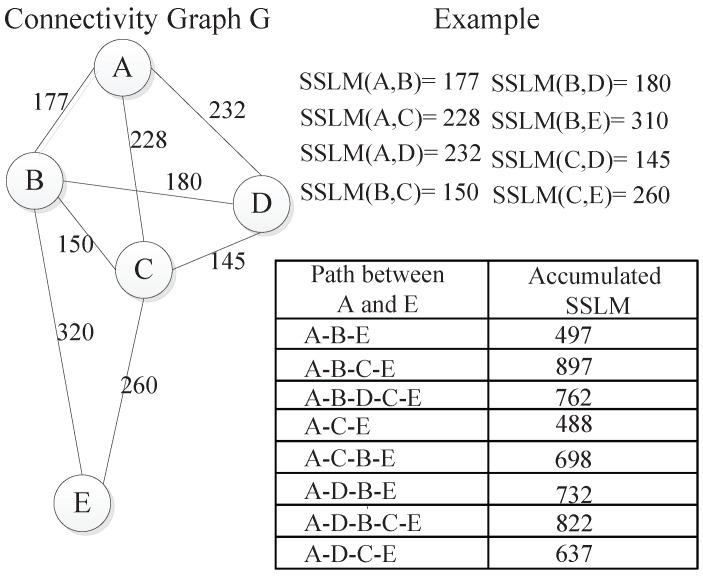
Distance estimation for non-neighboring nodes.

Taking the non-neighboring node pair (A,E) as an example, we first search all of the paths between them after obtaining the SSLM values of all one-hop neighboring nodes and then summarize SSLM values of every path. Finally, the smallest accumulated SSLM value is assigned as their relative distance, which is 488 in this example. Figure 3 illustrates the calculation process of the relative distance. On the right of the figure, all neighboring nodes’ SSLM values are listed, and all of the accumulated SSLM values between *A* and *E* are also shown. In all, for one-hop neighboring nodes *i* and *j*, the relative distance equals SSLM(i,j) computed with Formula (1). For non-neighboring nodes *i* and *j*, the relative distance is calculated as the smallest summation of the SSLM values along a path between them. Utilizing the above calculation method, we can finally get the relative distance of any node pair in the whole network.

In the implementation process, we can employ graph knowledge to solve the problem of the smallest accumulated SSLM as follows. Given the network connectivity graph *G*, we use the vertex set V(G) to denote all nodes in the network. Additionally, the edge set E(G) is used to indicate the distance of neighboring nodes. The weights of the edges are represented by SSLM values. Let the accumulated edge’s weight be the length of the path; the shortest path can then be solved by the Floyd–Warshall algorithm [35].

### 4.3. Node Position Estimation

Starting with the network connectivity information and RSSI values, we have already roughly estimated the relative distance between each pair of nodes by using the shortest path algorithm. Thus, a distance matrix can be constructed in which the value at the *i*-th row and the *j*-th column is the relative distance between nodes *i* and *j*. In the following, we can adopt many existing schemes to localize the nodes without changing their major design. For example, the distance matrix here is the smallest accumulated SSLM values instead of the shortest hop distances in MDS-MAP and shortest-path hops in DV-Hop, while the others remain the same.

Considering the requirements and characteristics of the ocean monitoring, the MDS technology is adopted to construct a relative map of the network from the calculated relative distance matrix, which can deduce node locations satisfying those estimated distances. Then, an “absolute map” can be obtained by scaling and rotating the relative map based on the physical coordinates of at least three anchors. There currently exist many types of MDS techniques to find a placement of the nodes in a two-dimensional space. Since one distance matrix is used in this paper, we only employ the classical metric MDS. It is the simplest case of MDS and tolerates error gracefully, due to the overdetermined nature of the solution. Moreover, the solution is closed-form, which is very suitable for an ocean sensor network consisting of a large numbers of nodes. The detailed description of MDS can be found in [36]. Here, we do not illustrate it further.

### 4.4. Algorithm Analysis

**Algorithm 1** SSL Node Localization Algorithm
**Input:**   RSS, Anchor, N; **Output:**   SSL;
1:ND=CalNeighboringDistance(RSS)
2:      %Calculate the relative distance of any neighboring nodes3:**for** i=1:N
4:   **for** j=i+1:N5:      $S_{ij}=ReferenceSet\left(S_{i},S_{j}\right)$6:      $\left(V_{i},V_{j}\right)=VectorGeneration\left(S_{i}j\right)$7:      $SSLM_{ij}=CalManha\left(Vi,Vj\right)$8:   **end**9:**end**10:ShortestND=CalNonneighboringDistance(ND)
11:      %Calculate the relative distance of non-neighboring nodes12:TempND=ND
13:ShortestND=ND
14:**for** k=1:N
15:   **for** i=1:N16:      **for** j=1:N17:         **if** TempND(i,j)≤(TempND(i,k)+TempND(k,j))18:            ShortestND(i,j)=TempND(i,j)19:         **else**20:            ShortestND(i,j)=TempND(i,k)+TempND(k,j)21:         **end**22:      **end**23:   **end**24:   TempND=ShortestND25:**end**26:SSL=MDSrealLocation(ShortestND,Anchor,tol)
27:      % Convert the relative positions to absolute locations, where tol is the absolute error tolerance


Algorithm 1 illustrates the whole localization procedure. The relative distances are firstly calculated through comparing the similarity of RSSI vectors. Given the anchors’ coordinates, each node’s location is estimated by scaling and rotating the relative distance map based on the MDS technology. The computational complexity of constructing the reference set is O(MlogM+NlogN), where *M* and *N* are the numbers of neighbors of two nodes, respectively. Additionally, the computational complexity of estimating the relative distance between two neighboring nodes is only O(1). From the localization procedure, we can see that the involvement of the RSSI similarity for distance estimation introduces little additional cost, since the shortest path calculation or the MDS technology is also used in traditional location methods.

Regarding the communication cost in the localization process, the additional overhead is the exchanging of RSSI values among one-hop nodes. Note that it only requires neighboring nodes to exchange the RSSI value instead of the network flooding. Additionally, the RSSI values can be encapsulated in the messages during the network initialization phase. Hence, the proposed design is energy efficient and low cost. After getting the RSSI values from the neighbors, the calculation of SSLM can be implemented at each node or by a localization server deployed at the seashore. Obviously, it costs few time and space resources to calculate the Manhattan distance. Therefore, the proposed algorithm does not affect the scalability of the system and falls into the connectivity-based localization methods.

## 5. Experiments

In this section, we describe the performance gain obtained from the SSL design for ocean sensor networks with two experiment scenarios.

### 5.1. Experiment Setup

**Figure 4:**
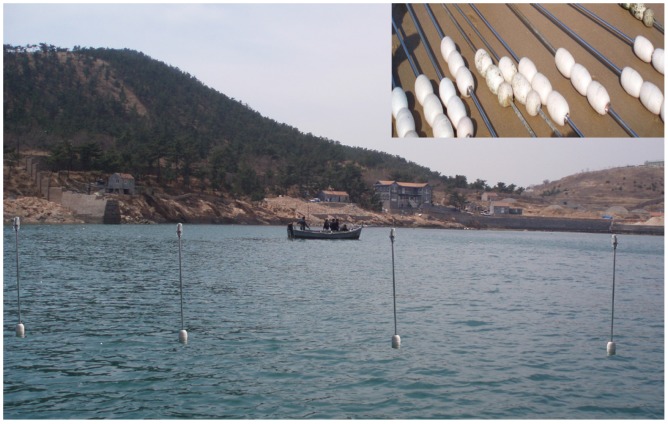
Experiments for the ocean monitoring.

Generally, there are two types of WSN applications for ocean surface monitoring. The first one is the sea route monitoring, in which nodes are often deployed as the zonal network to monitor the passing ships. The other scenario is the monitoring of the ocean environment, in which several nodes are deployed on the sea surface to monitor the temperature, light, and so on. The experiments of two scenarios will both be conducted in this paper. Figure 4 shows the experiment scenario. To protect the nodes from seawater and sunshine, the sensor is encapsulated into a transparent plastic bottle. Additionally, each node is left 40 inches above the sea surface with 3 lightweight supporting foam balls.

In the experiments, every sensor broadcasts 80 packets with the radio sending power of 0 dBm. The technologies of carrier sensing and back-off time are adopted to avoid collision. Each packet contains the sender’s node ID and a sequence number of the packet. If the received signal strength is larger than -100 dBm (the receiver sensitivity threshold), the corresponding RSSI value, the sender’s ID and the sequence numbers of the packet will be written into its flash memory or the MAC frame will be abandoned. In order to eliminate the effect of the radio path loss, multi-path, hardware discrepancies, and so on, each node will choose the median one from the 80 RSSI values as the final RSSI value when the broadcasting is ended.

As is well known, DV-Hop estimates the expected one-hop physical distance based on the average hop distance and hops among anchors. Because the deployed network in this paper has at most 3 hops, the direct using of DV-Hop is not fair and suitable. Here, we employ the “MDS-Hop” localization method, which is described as below. For each sensor, it estimates its distance to another node with the number of hops and the expected hop distance. After obtaining the distance matrix using DV-Hop, we can use MDS to localize the node. Similarly, “MDS-SSLM” and “MDS-SSLE” are the corresponding localization methods embedded with SSLM and SSLE, respectively. In the following, we will describe and discuss the localization accuracy of the three methods.

### 5.2. Zonal Network

**Figure 5:**
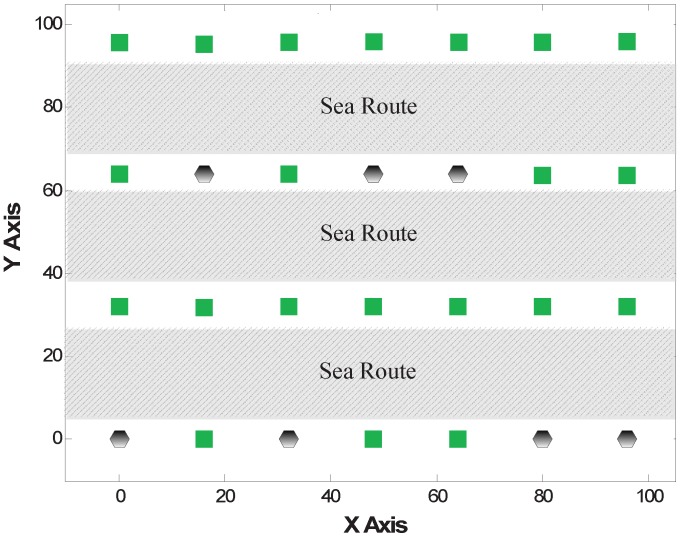
Zonal network.

In this experiment, 28 nodes are deployed to be a zonal network for monitoring three sea routes. Additionally, there are 7 nodes on each side of the sea route. As shown in Figure 5, 7 nodes are selected as anchors and depicted in black hexagons, while the green squares are the deployed positions of general nodes. The distance between two adjacent nodes along the X-axis is about 16 feet, while the distance of adjacent nodes along the Y-axis is about 32 feet.

#### 5.2.1. Distance Correlation in a Zonal Network

The effectiveness of the estimated relative distance will first be evaluated. Three relative distances are compared, as described below. The first one is traditional hop-based distance, in which the number of hops between any two nodes is regarded as their relative distance. The other two relative distances are the SSLM and SSLE mentioned in this paper.

**Figure 6:**
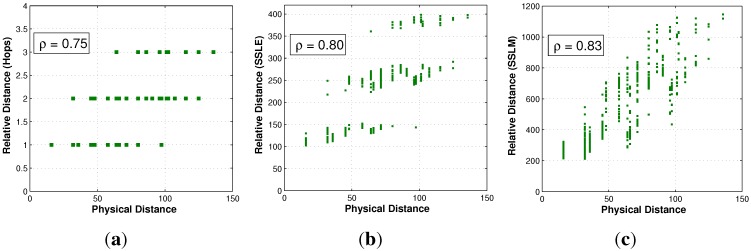
Distance correlation comparison. (**a**) Correlation between hop and physical distance; (**b**) correlation between SSL with the Euclidean distance (SSLE) and physical distance; (**c**) correlation between SSL with the Manhattan distance (SSLM) and physical distance.

The effectiveness is determined by the correlation between their values and the physical distance. A higher correlation coefficient implies that the corresponding relative distance reflects the real distance better. The correlation coefficient for the neighboring nodes is zero in the hop-based distance, because all one-hop node pairs have the same hop distance. SSLE and SSLM are surely superior to hop-based distance for proximity expression. Thus, we just give the correlation coefficient comparison between relative distance and physical distance from the view point of the whole network, as shown in Figure 6. The X-axis is the physical distance between two nodes in all of the sub-figures, while the Y-axis is the hop-based distance, SSLE and SSLM, respectively. The experiment data shown in Figure 6c confirm that SSLM (with the correlation coefficient ρ=0.83) provides a better resolution than SSLE and hop-based distance. From the results, we can see that the effective use of the RSSI value can be indeed a good heuristic indicator for physical distance.

#### 5.2.2. Comparison of Localization Results

**Figure 7:**
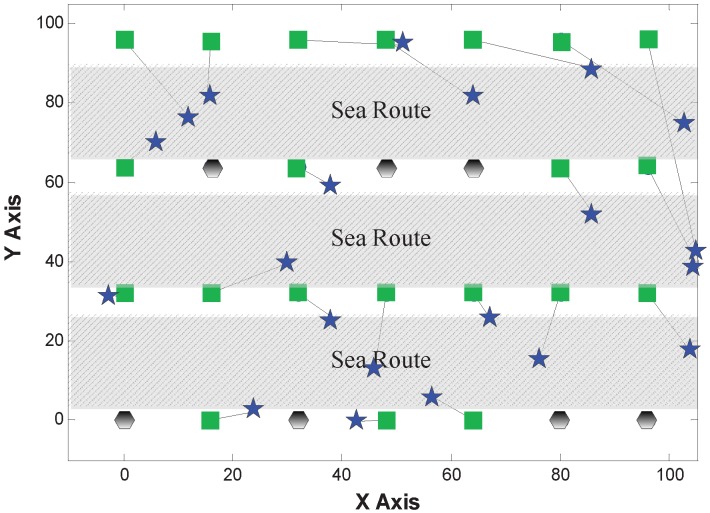
Localization results of multi-dimensional scaling (MDS)-Hop.

**Figure 8:**
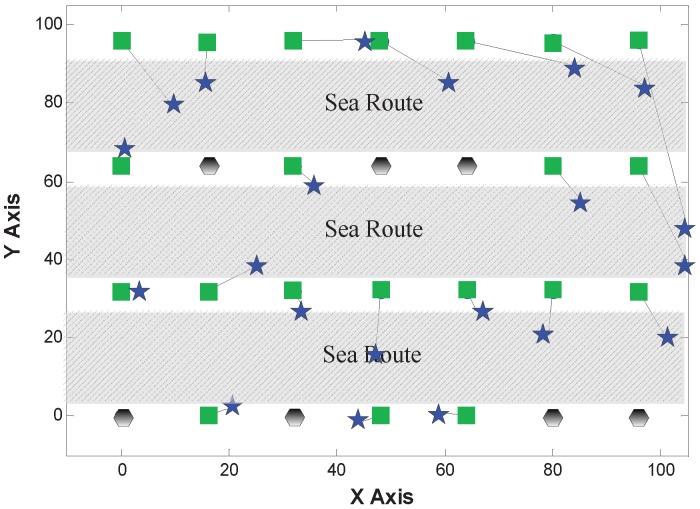
Localization results of MDS-SSLE.

**Figure 9:**
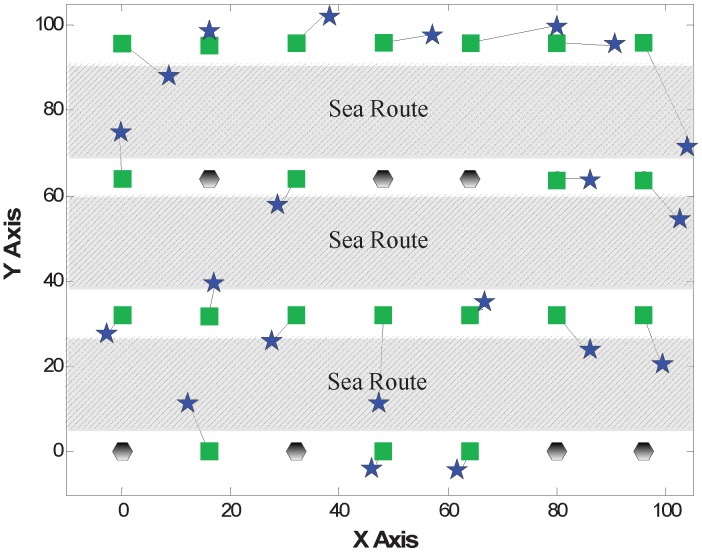
Localization results of MDS-SSLM.

Figure 7, Figure 8 and Figure 9 show the localization results of MDS-Hop, MDS-SSLM and MDS-SSLE, respectively. In the figures, blue stars are used to depict the estimated positions, while the black lines represent the offset of node locations. This result confirms that the MDS-SSLM method offers a better resolution than MDS-SSLE and MDS-Hop.

The localization error is defined as the distance from the real position of a node to its estimated location in this paper. Table 1 shows the maximum and median localization errors from the results in the above figures for all three methods. We can clearly observe that the MDS-SSLM method has smaller errors than MDS-SSLE and MDS-Hop. Specifically, the maximum and mean errors of MDS-SSLM get reduced by 53% and 37% compared to MDS-Hop. Compared to MDS-SSLE, the maximum and median errors are reduced by 48% and 49%. This result confirms that the Manhattan-based distance estimation has better accuracy and stability than the Euclidean-based version.

**Table 1:** Error statistics for a zonal network.

Error	MDS-Hop	MDS-SSLE	MDS-SSLM
Median Error	4.9569	6.1568	3.1087
Max Error	16.0075	14.5258	7.5756

#### 5.2.3. Impact of Anchor Density in a Zonal Network

**Figure 10:**
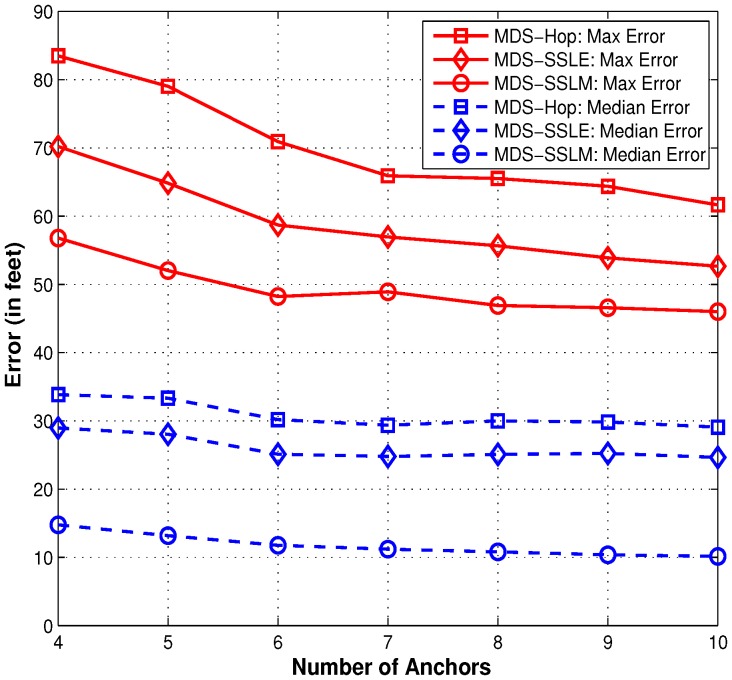
Statistical comparison of the three localization methods.

In order to eliminate the possible bias caused by anchor selection, we randomly pick different numbers of anchors, from 4 to 10, and try each for 1500 runs. From Figure 10, we can obviously see that more anchors help improve the localization accuracy for all three methods. Note that MDS-SSLM is more effective than MDS-Hop. By introducing the similarity of RSSI values, the localization error gets reduced constantly by around 20% for MDS-Hop.

### 5.3. Non-Regular Network

In order to verify the universality of the SSL design, we conduct the second experiment, which is a non-regular network with 28 nodes. The network covers an area of about 100×100 square feet with 7 selected anchors.

#### 5.3.1. Distance Correlation in a Non-Regular Network

**Figure 11:**
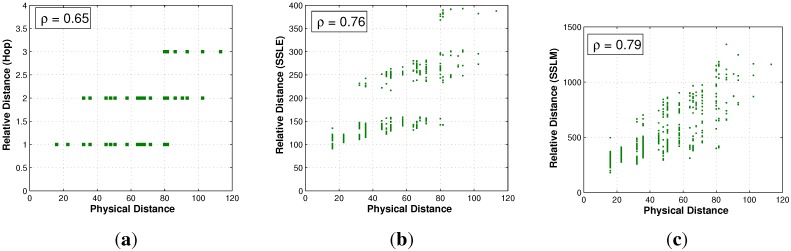
Distance correlation comparison. (**a**) Correlation between hop and physical distance; (**b**) correlation between SSLE and physical distance; (**c**) correlation between SSLM and physical distance.

Here, the correlations between hop-based distance, SSLE, SSLM and physical distance are evaluated for the non-regular network. In all of the figures, the X-axis is the physical distance between two nodes, and the Y-axis is the hop-based distance, SSLE, SSLM, respectively. Figure 11a displays the relation between the hop distance and physical distance. Similarly, Figure 11b,c plots the accumulated SSLE and SSLM against physical distance for all node pairs. Comparing these three figures, SSLM provides better resolution with the correlation coefficient ρ=0.79. Specifically, a physical distance can only be mapped to an integer hop distance in a discrete manner in Figure 11a, while the mapping is continuous in Figure 11c. Obviously, the continuous mapping can better reflect the physical distance relationship.

#### 5.3.2. Comparison of Localization Results

**Figure 12:**
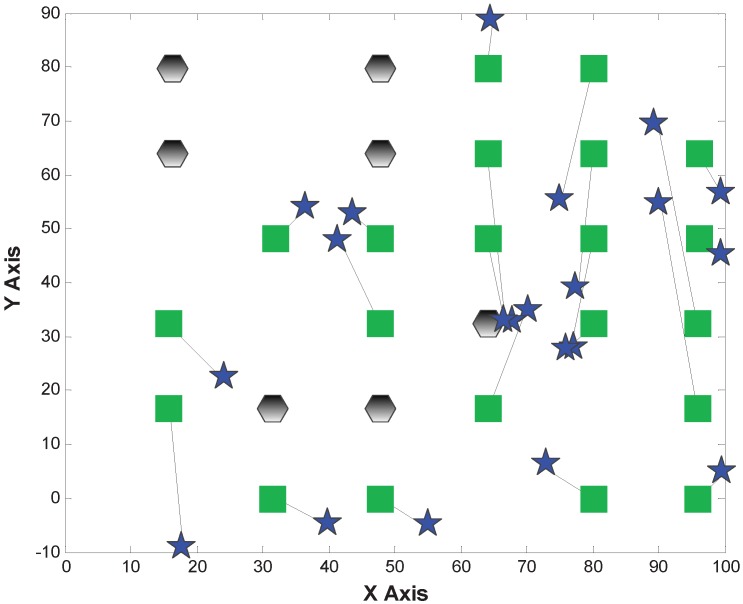
Localization results of MDS-Hop.

**Figure 13:**
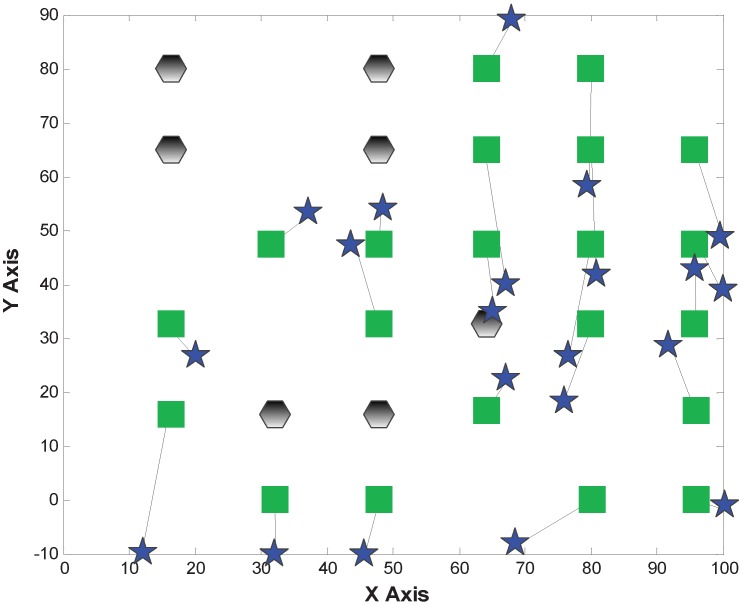
Localization results of MDS-SSLE.

**Figure 14:**
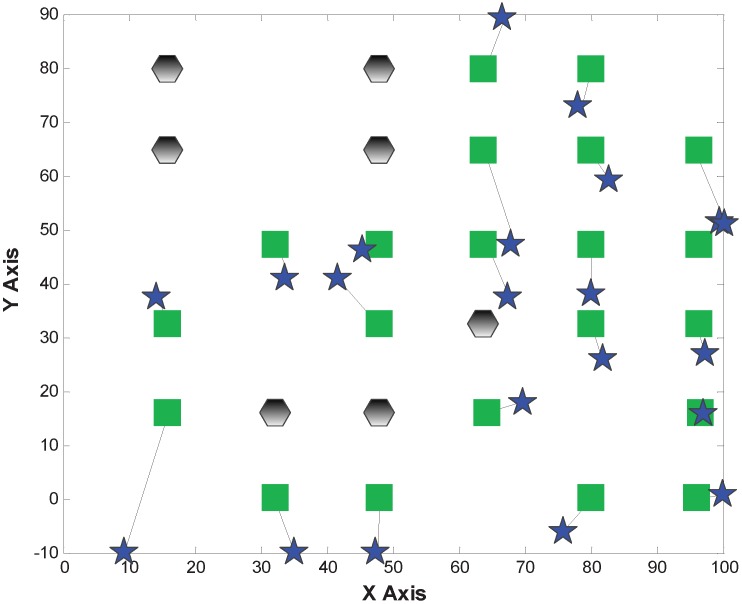
Localization results of MDS-SSLM.

Figure 12, Figure 13 and Figure 14 depict the localization results from MDS-Hop, MDS-SSLM and MDS-SSLE. In the above figures, the blue star is the estimated location, and the line segment is the offset between real and estimated positions. Similarly, from the three figures, we can see that MDS-SSLM also gives better localization accuracy than others for a non-regular network.

The localization errors of the three methods are listed in Table 2. From the table, we can observe that the maximum and median errors of MDS-SSLM get reduced obviously compared to the MDS-Hop, which are 51% and 63%, respectively.

**Table 2:** Error statistics for a non-regular network.

Error	MDS-Hop	MDS-SSLE	MDS-SSLM
Median Error	6.0175	4.4897	2.2011
Max Error	18.7441	15.7196	9.1300

#### 5.3.3. Impact of Anchor Density in a Non-Regular Network

The above results are obtained based on the given seven anchors. In order to evaluate the impact of anchor placement and anchor number, we further conduct the accuracy evaluation for different numbers of anchors in a non-regular network, in which the anchors are randomly selected and 1500 rounds are performed to achieve a fair comparison.

**Figure 15:**
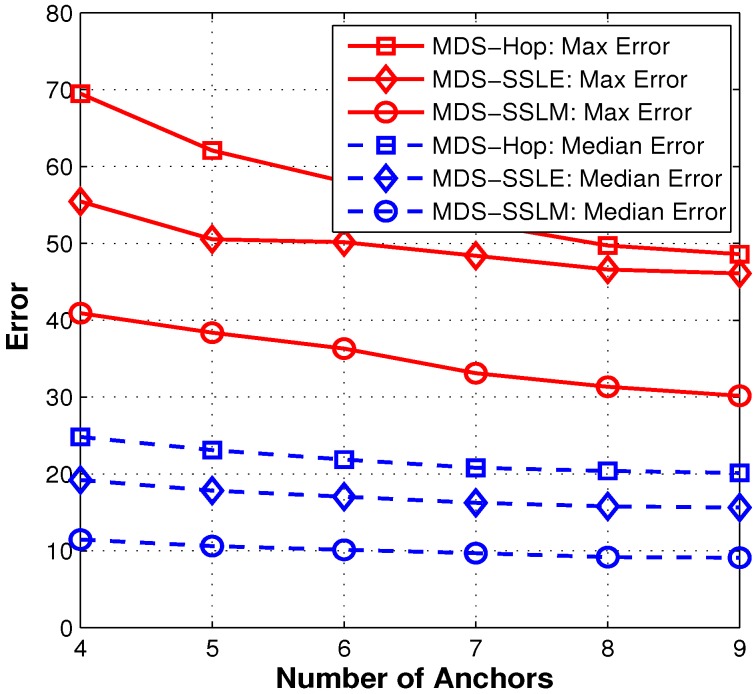
Statistical comparison of the three localization methods.

As expected, Figure 15 shows that more anchors help improve the localization accuracy for all methods. It is noticed that the MDS-SSLM method is more effective than adding extra anchors, especially after 7 anchors when curves become flat. This characteristic can help to reduce the number of deployed anchors and improve the system robustness under a certain localization requirement.

## 6. Conclusions

In this paper, we propose a novel localization scheme for wireless sensors deployed on the sea surface, which is based on the similarity comparison of the RSSI values. The estimation of node location is obtained only by means of the received signal strength information, which is energy efficient, robust and low cost. Hence, the proposed design is very practical for ocean monitoring. Experimental results reveal that the proposed algorithm offers better localization accuracy than the traditional schemes. Considering the good results obtained, we have decided to broaden the algorithm for constructing the large-scale experiments in a real ocean environment in the future.

## References

[1] Xu G.B., Shen W.M., Wang X.B. (2014). Applications of wireless sensor networks in marine environment monitoring: A survey. Sensors.

[2] Liu K.B., Yang Z., Li M., Guo Z.W., Guo Y., Hong F., Yang X.H., He Y., Feng Y., Liu Y.H. (2010). Ocean sense: Monitoring the sea with wireless sensor networks. ACM SIGMOBILE Mob. Comput. Commun. Rev..

[3] Liu Y.H., Liu K.B., Li M. (2010). Passive diagnosis for wireless sensor networks. IEEE/ACM Trans. Netw..

[4] Xu B., Sun G.D., Yu R., Yang Z. (2013). High-accuracy TDOA-based localization without time synchronization. IEEE Trans. Parallel Distrib. Syst..

[5] Bahl P., Padmanabhan V.N. RADAR: An in-building RF-based user location and tracking system. Proceedings of the IEEE International Conference on Computer Communications (INFOCOM 2000).

[6] Niculescu D., Nath B. Ad hoc positioning system (APS) using AOA. Proceedings of the IEEE International Conference on Computer Communications (INFOCOM 2003).

[7] Savvides A., Han C.-C., Strivastava M.B. Dynamic fine-grained localization in ad-hoc networks of sensors. Proceedings of the 7th Annual International Conference on Mobile Computing and Networking (MOBICOM 2001).

[8] Cheng X.Z., Thaeler A., Xue G.L., Chen D.C. TPS: A time-based positioning scheme for outdoor wireless sensor networks. Proceedings of the IEEE International Conference on Computer Communications (INFOCOM 2004).

[9] Priyantha N.B., Chakraborty A., Balakrishnan H. The Cricket location-support system. Proceedings of the 6th Annual International Conference on Mobile Computing and Networking (MOBICOM 2000).

[10] Whitehouse K., Karlof C., Culler D. (2007). A practical evaluation of radio signal strength for ranging-based localization. ACM Mob. Comput. Commun. Rev..

[11] Lederer S., Wang Y., Gao J. Connectivity-based localization of large scale sensor networks with complex shape. Proceedings of the IEEE International Conference on Computer Communications (INFOCOM 2008).

[12] Nagpal R., Shrobe H., Bachrach J. Organizing a global coordinate system from local information on an ad hoc sensor network. Proceedings of the 2nd International Workshop on Information Processing in Sensor Networks (IPSN 2003).

[13] Savarese C., Rabaey J.M., Langendoen K. Robust positioning algorithms for distributed ad-hoc wireless sensor networks. Proceedings of the General Track of the Annual Conference on USENIX Annual Technical Conference.

[14] He T., Huang C.D., Blum B.M., Stankovic J.A. Range-free localization schemes for large-scale sensor networks. Proceedings of the 9th Annual International Conference on Mobile Computing and Networking (MOBICOM 2003).

[15] Doherty L., Pister K.S.J., El Ghaoui L. Convex position estimation in wireless sensor networks. Proceedings of the IEEE International Conference on Computer Communications (INFOCOM 2001).

[16] Bulusu N., Heidemann J., Estrin D., Tran T. (2004). Self-configuring localization systems: Design and experimental evaluation. ACM Trans. Embed. Comput..

[17] Zhong Z.G., He T. (2012). Node localization with uncontrolled events. ACM Trans. Embed. Comput..

[18] Stoleru R., He T., Stankovic J.A., Luebke D. A high-accuracy, low cost localization system for wireless sensor networks. Proceedings of the 3rd International Conference on Embedded Networked Sensor Systems (SENSYS 2005).

[19] Jeong J., Guo S., He T., Du D.H.C. (2011). Autonomous passive localization algorithm for road sensor networks. IEEE Trans. Comput..

[20] Guo Z.W., Chen P.P., Zhang H., Jiang M.X., Li C.R. (2012). IMA: An integrated monitoring architecture with sensor networks. IEEE Trans. Instrum. Meas..

[21] Wu C.S., Yang Z., Zhou Z.M., Qian K., Liu Y.H., Liu M.Y. PhaseU: Real-time LOS identification with WiFi. Proceedings of the IEEE International Conference on Computer Communications (INFOCOM 2015).

[22] Wen Y.T., Tian X.H., Wang X.B., Lu S.W. Fundamental limits of RSS fingerprinting based indoor localization. Proceedings of the IEEE International Conference on Computer Communications (INFOCOM 2015).

[23] Chen P.P., Zhong Z.G., He T. Bubble trace: Mobile target tracking under insufficient anchor coverage. Proceedings of the IEEE International Conference on Distributed Computing Systems (ICDCS 2011).

[24] Goldenberg D.K., Bihler P., Cao M., Fang J., Anderson B.D.O., Morse A.S., Yang Y.R. Localization in sparse networks using sweeps. Proceedings of the 12th Annual International Conference on Mobile Computing and Networking (MOBICOM 2006).

[25] Yang Z., Liu Y.H. Quality of trilateration: Confidence-based iterative localization. Proceedings of the IEEE International Conference on Distributed Computing Systems (ICDCS 2008).

[26] Cheng X.Z., Shu H.N., Liang Q.L., Du D.H.-C. (2008). Silent positioning in underwater acoustic sensor networks. IEEE Trans. Veh. Technol..

[27] Chang H.-L., Tian J.-B., Lai T.-T., Chu H.-H., Huang P. Spinning beacons for precise indoor localization. Proceedings of the 6th ACM Conference on Embedded Networked Sensor Systems (SENSYS 2008).

[28] Zhong Z.G., He T. MSP: Multi-sequence positioning of wireless sensor nodes. Proceedings of the 5th ACM Conference on Embedded Networked Sensor Systems (SENSYS 2007).

[29] Wang C., Xiao L. Locating sensors in concave areas. Proceedings of the IEEE International Conference on Computer Communications (INFOCOM 2006).

[30] Niculescu D., Nath B. (2003). DV based positioning in ad-hoc networks. Telecommun. Syst. Model. Anal. Des. Manag..

[31] Shang Y., Ruml W., Zhang Y., Fromherz M.P.J. Localization from mere connectivity. Proceedings of the 4th ACM International Symposium on Mobile Ad Hoc Networking and Computing (MOBIHOC 2003).

[32] Chen P.P., Ma H.L., Gao S.W., Huang Y. (2015). Modified Extended Kalman Filtering for Tracking with Insufficient and Intermittent Observations. Math. Probl. Eng..

[33] Zhong Z.G., He T. (2011). RSD: A metric for achieving range-free localization beyond connectivity. IEEE Trans. Parallel Distrib. Syst..

[34] Xi W., He Y., Liu Y.H., Zhao J.Z., Mo L.F., Yang Z., Wang J.L., Li X.Y. Locating sensors in the wild: Pursuit of ranging quality. Proceedings of the 8th ACM Conference on Embedded Networked Sensor Systems (SENSYS 2010).

[35] Cormen T.H., Leiserson C.E., Rivest R.L., Stein C. (2003). Introduction to Algorithms.

[36] Greenacre M.J. (1984). Theory and Applications of Correspondence Analysis.

